# PhoP- and GlnR-mediated regulation of *metK* transcription and its impact upon S-adenosyl-methionine biosynthesis in *Saccharopolyspora erythraea*

**DOI:** 10.1186/s12934-022-01846-w

**Published:** 2022-06-18

**Authors:** Jin-Feng Pei, Yu-Xin Li, Hao Tang, Wenping Wei, Bang-Ce Ye

**Affiliations:** 1 Collaborative Innovation Center of Yangtze River Delta Region Green Pharmaceuticals, College of Pharmaceutical Sciences, Institute of Engineering Biology and Health, Zhejiang University of Technology, Hangzhou, Zhejiang China; 2 Lab of Biosystems and Microanalysis, State Key Laboratory of Bioreactor Engineering, Institute of Engineering Biology and Health, East China University of Science and Technology, Shanghai, China

**Keywords:** Erythromycin A, Transcriptional regulation, PhoP, GlnR, SAM

## Abstract

**Background:**

Erythromycin A (Er A) has a broad antibacterial effect and is a source of erythromycin derivatives. Methylation of erythromycin C (Er C), catalyzed by *S*-adenosyl-methionine (SAM)-dependent *O*-methyltransferase EryG, is the key final step in Er A biosynthesis. Er A biosynthesis, including EryG production, is regulated by the phosphate response factor PhoP and the nitrogen response factor GlnR. However, the regulatory effect of these proteins upon *S*-adenosyl-methionine synthetase (MetK) production is unknown.

**Results:**

In this study, we used bioinformatics approaches to identify *metK* (SACE_3900), which codes for *S*-adenosyl-methionine synthetase (MetK). Electrophoretic mobility shift assays (EMSAs) revealed that PhoP and GlnR directly interact with the promoter of *metK*, and quantitative PCR (RT-qPCR) confirmed that each protein positively regulated *metK* transcription. Moreover, intracellular SAM was increased upon overexpression of either *phoP* or *glnR* under phosphate or nitrogen limited conditions, respectively. Finally, both the production of Er A and the transformation ratio from Er C to Er A increased upon *phoP* overexpression, but surprisingly, not upon *glnR* overexpression.

**Conclusions:**

Manipulating the phosphate and nitrogen response factors, PhoP and GlnR provides a novel strategy for increasing the yield of SAM and the production of Er A in *Saccharopolyspora erythraea *.

**Supplementary Information:**

The online version contains supplementary material available at 10.1186/s12934-022-01846-w.

## Background

*Saccharopolyspora erythraea* has been used for the production of erythromycin A (Er A), a broad-spectrum macrolide antibiotic that is effective against pathogenic gram-positive bacteria [1, 2]. In recent years, Er A has been in vast demand owing to its good antibacterial effect and the therapeutic potential of its semi-synthetic derivatives [3]. Erythromycin production is a world-wide, large-scale industrial process, and methylation of erythromycin C (Er C) to Er A is one of the main manufacturing bottlenecks, therefore, it is necessary that methods used to promote the production and purity of Er A, such as regulatory strategy, are improved to better meet market demand. Er A biosynthesis involves the formation of a macrolide intermediate, 6-deoxyerythronolide B (6-DEB), and its subsequent modifications [3–5]. These modifications include hydroxylation of the C-12 site at the aglycone and *O*-methylation of the C-3 site at the macrose, which are catalyzed by the P450 hydroxylase (EryK) and S-adenosyl-methionine (SAM)-dependent O-methyltransferase (EryG), respectively [6–8]. The synthesis of Er A is accompanied by the synthesis of Er C and erythromycin B (Er B), which can affect the yield and purity of Er A during fermentation [3, 9]. Methylation is the key and final step in the synthesis of Er A from Er C, which is catalyzed by the EryG. As a catalytic substrate for EryG, increasing the supply of SAM is conducive to increasing the synthesis of antibiotics requiring methylation modification [10–16]. In addition, SAM plays an important role in intracellular processes and a variety of biosynthetic machineries; it is an active methyl donor and regulatory factor [17, 18]. Therefore, enhancing *S*-adenosyl-methionine synthetase (MetK) expression is a common strategy for improving antibiotic synthesis [10–16]. In addition, it is well known that the expression of gene clusters related to erythromycin synthesis is regulated by the phosphate-sensing factor PhoP and the nitrogen-sensing factor GlnR [19, 20]. Interestingly, the EryG gene exists in the middle of the erythromycin synthesis gene cluster in *S. erythraea* and is regulated by PhoP [19]. However, to date, the mechanism by which PhoP and GlnR regulate the MetK-encoding gene *metK* and its affect upon the supply of intracellular SAM has not yet been resolved. Investigating the regulatory relationship between PhoP and GlnR and the expression of *metK* is conducive to enhancing the understanding of the nutritional response of actinomycetes, the supply of SAM, and the synthesis of methylation-modified antibiotics [1, 12, 19, 21].

PhoP and GlnR have been reported to respond to phosphate and nitrogen stress and to regulate antibiotic production in species such as in *Streptomyces coelicolor* [22], *S. erythraea* [19], and *Streptomyces natalensis* [23]. PhoP- and GlnR-mediated regulation of phosphate and nitrogen metabolism affects the expression of genes related to antibiotic synthesis, precursor supply, post-modification of antibiotics, and so on. PhoP senses the change in phosphorus signal and directly or indirectly regulates the biosynthesis of undecylprodigiosin in *S. coelicolor* [22], actinorhodin in *Streptomyces lividans* [24], and polyene macrolide pimaricin in *S. natalensis* [23]. In *S. erythraea*, the biosynthesis of Er A is also sensitive to phosphate concentration in the growth media [19]. Phosphate limitation strongly induces expression of the *ery* gene cluster and further promotes the biosynthesis of Er A [19]. Similarly, GlnR also regulates antibiotic biosynthesis in *Streptomyces* [20, 25]. In *S. coelicolor* A3(2), the deletion of *glnR* led to a remarkable increase in actinorhodin production [25]. The same phenomenon was also found in the rifamycin producer *Amycolatopsis mediterranei* [26] and erythromycin producer *S. erythraea* [20]. In summary, since PhoP and GlnR have a relatively general regulatory effect on antibiotic synthesis gene clusters, the question becomes, do they have a similar transcriptional regulation effect on *metK* and the supply of SAM?

In this work, we performed a series of systematic studies to analyze the PhoP- and GlnR-mediated regulation of *metK* expression. EMSAs confirmed that PhoP and GlnR interact with the operator interval of the *metK* gene promoter. A transcription difference further verified that *metK* expression level was influenced by PhoP and GlnR, and confirmed the direct regulatory mechanism of PhoP and GlnR on *metK* expression. Finally, by assessing Er A production we provide evidence that PhoP and GlnR-mediated regulation of *metK* expression might be an effective target for influencing the synthesis of engineered methyl-modified antibiotics in *actinobacteria*.

## Results

### PhoP and GlnR interact with the regulatory region of SACE_3900 gene encoding S-adenosylmethionine synthetase

**Fig. 1:**
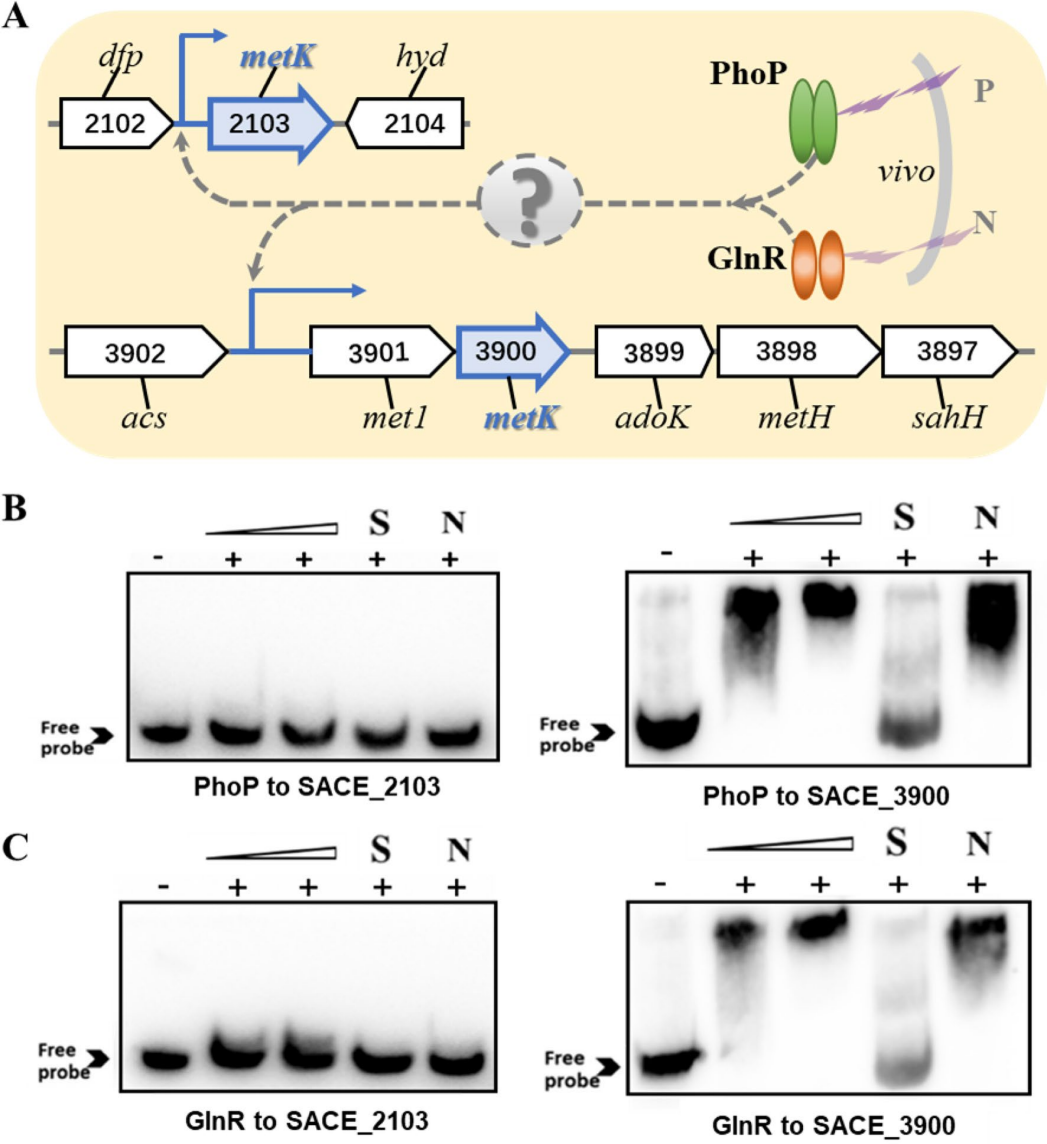
Interaction between PhoP or GlnR and the promoter region of putative *metK* genes. **A** Schematic representation of the genetic positioning of putative *metK* genes in *S. erythraea*, and the possible regulatory effect of PhoP and GlnR. **B** Interaction assessment for His_6_-PhoP and the upstream promoter region of SACE_2103 (left) and SACE_3900 (right). **C** Interaction assessment for His_6_-GlnR and the upstream promoter region of SACE_2103 (left) and SACE_3900 (right). Concentration of His_6_-tagged PhoP and GlnR protein was (Lane 1, 0; Lane 2, 0.2; and Lane 3, 0.4 μM). Arrow heads show the free probes without bound protein. Unlabeled specific probe, S; non-specific competitor DNA, N; DNA/pantothenate metabolism flavoprotein, *dfp*; hydrolase (secreted trypsin-like serine protease), *hyd*; S-adenosyl-L-homocysteine hydrolase, *sahH*; 5-methyltetrahydrofolate–homocysteine methyltransferase, *metH*; adenosine kinase, *adoK*; S-adenosylmethionine synthetase, *metK*; glycine sarcosine N-methyltransferase, *met1*; acetyl-CoA synthetase (AMP-dependent synthetase/ligase), *acs.*

Previous studies have shown that PhoP and GlnR have a regulatory effect on erythromycin synthesis and the expression of central metabolism-related genes [19, 20, 27]. To investigate the influence of PhoP and GlnR on the expression of the SAM-donor for the erythromycin side chain methyl group, it was first necessary to identify the *metK* in *S. erythraea* [17]. Base on the annotation from the KEGG database and genome sequence analysis, the genome of *S. erythraea* possesses two putative *metK* genes (SACE_2103 and SACE_3900) [17]. The genes shared high sequence similarity at the amino acid level (approximately 88.9%) (Additional file 1: Fig. S1). Therefore, the promoter regions of SACE_2103 and SACE_3900 were selected for in vitro experiments to investigate the binding effects of PhoP and GlnR (Fig. 1A). In this study, a classic EMSAs experiment was used to determine whether PhoP and GlnR interact with the promoter region of each putative target gene. The 5-Biotin-labeled SACE_2103 and SACE_3900 DNA probes were mixed with purified His_6_-tagged PhoP or GlnR. For SACE_3900, shifted bands were found in the labeled SACE_3900 probe lane for both PhoP and GlnR (Fig. 1B, C). Unlabeled specific probe (S) and non-specific competitor DNA (N) were included to confirm the specificity of the binding. These data verify that the PhoP and GlnR proteins specifically bind to the promoter region of SACE_3900, which is consistent with our bioinformatic prediction. In contrast, neither His_6_-tagged PhoP nor GlnR caused a band shift when incubated with SACE_2103, which suggests that these proteins do not interact with the promoter of this gene. Utilizing MAST/MEME tools and previously reported conserved binding sequence [2], no potential PhoP or GlnR binding motifs in the upstream promoter region of SACE_2103 were detected.

**Fig. 2:**
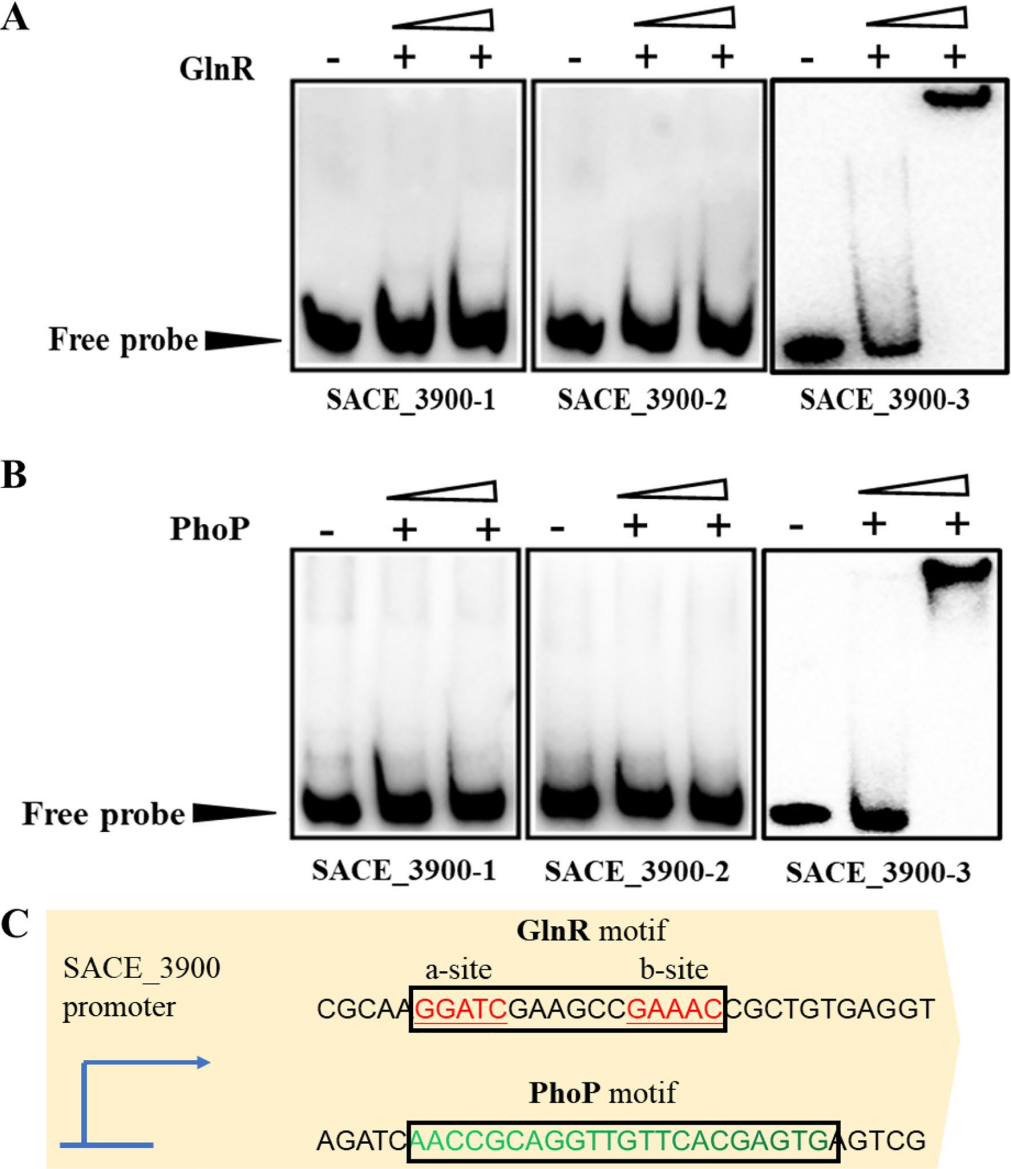
Analysis of the PhoP- and GlnR-binding region for the SACE_3900 promoter. **A** 5-biotin labeled SACE_3900-1/-2/-3 DNA fragments were reaction with His_6_-tagged GlnR and were analyzed using EMSA. **B** 5-biotin labeled SACE_3900-1/-2/-3 DNA fragments were reaction with His_6_-tagged PhoP and analyzed using EMSA. **C** Speculated binding motif of GlnR and PhoP protein in the SACE_3900 upstream promoter. His_6_-tagged PhoP (0, 0.3 and 0.6 μM) and GlnR (0, 0.4 and 0.8 μM) were added in reaction system. Arrowheads show the free probes without bound protein

EMSA_S_ results showed that regulators PhoP and GlnR specifically bind to the upstream promoter region of SACE_3900 (Fig. 1B, C). To precisely determine the PhoP and GlnR binding positions in the SACE_3900 promoter region, the upstream promoter region of SACE_3900 was divided into three sections (referred to as SACE_3900-1/-2/-3) (Additional file 1: Fig. S2) and each DNA fragment was mixed with various concentrations of His_6_-tagged PhoP or GlnR. SACE_3900-3 was found to harbor predicted PhoP and GlnR binding sites (Fig. 2A, B). The sectioned EMSAs further suggest that PhoP and GlnR possibly regulate SACE_3900 expression via specific interaction with the SACE_3900-3 region. Furthermore, putative GlnR-binding (Fig. 2C, a-site: GGATC; b-site: GAAAC) and PhoP-binding motifs (Fig. 2C, GTTCACGAGTG), were validated using MEME prediction software [28]. In summary, these results demonstrated that PhoP and GlnR have binding boxes in the promoter region of SACE_3900 and have potential regulatory functions for its expression.

### PhoP and GlnR positively regulate the transcription of SACE_3900

**Fig. 3:**
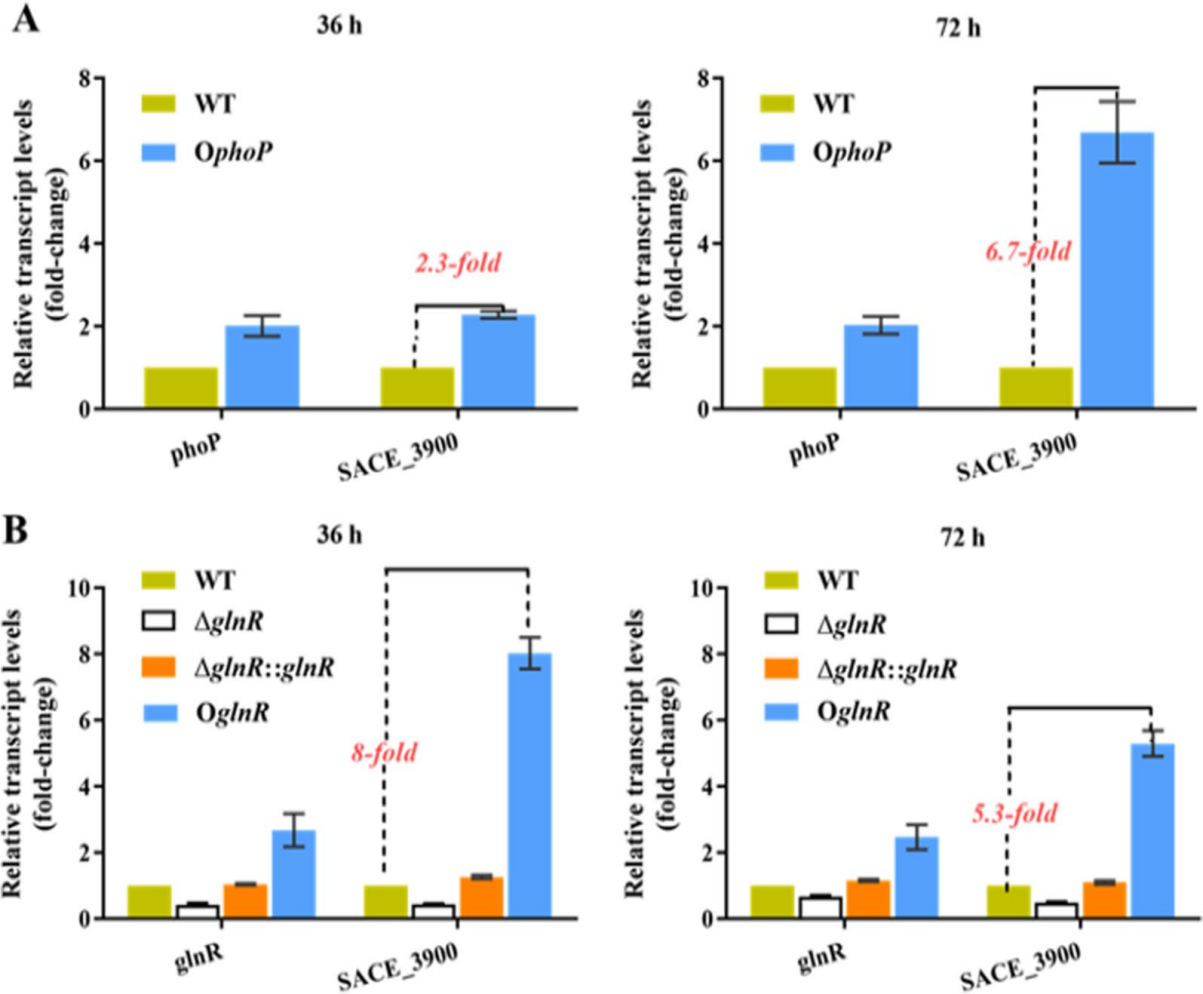
PhoP and GlnR positively regulate the expression of SACE_3900. **A** RT-qPCR analysis of *phoP* and SACE_3900 in WT and O*phoP* following 36 h or 72 h of growth. **B** Gene transcription of *glnR* and SACE_3900 in WT, Δ*glnR*, Δ*glnR*::*glnR*, O*glnR* strain at 36 and 72 h. The WT and O*phoP* were grown in phosphate-limiting Evans (40 μM KH_2_PO_4_ and C and N as basic), while WT, Δ*glnR*, Δ*glnR*::*glnR* and O*glnR* were grown in nitrogen-limiting Evans [1 mM (NH_4_)_2_SO_4_ and C and P as basic]. Error bars indicate standard deviations from three independent biological replicates

It has been reported that exogenous addition of SAM [13] or overexpression of the *metK* markedly enhances the production of antibiotics [12]. The specific binding sites of PhoP and GlnR on the SACE_3900 promoter were confirmed in vitro (Figs. 1 and 2). To determine if the interaction between PhoP or GlnR and the SACE_3900 promoter influences gene expression, we further investigated the transcription of SACE_3900 in liquid culture. To assess the effect of PhoP, the wild-type *S. erythraea* strain (WT), and the *phoP* overexpression strain (O*phoP*) were cultivated in phosphate-limiting modified Evans medium (see “Materials and methods” section). To assess effect of GlnR, WT, the *glnR* deletion strain (Δ*glnR*), the *glnR* complemented strain (Δ*glnR*::*glnR*), and the *glnR* overexpression strain (O*glnR*) were cultivated in nitrogen-limiting Evans (see “Materials and methods” section) [19]. The quantitative reverse transcription polymerase chain reaction (RT-qPCR) results showed that the expression of SACE_3900 in O*phoP* was upregulated 2.3- and 6.7-fold at 36 h and 72 h, respectively, when compared to WT (Fig. 3A). The observations demonstrated that PhoP specifically bound to the promoter region of SACE_3900 (Fig. 1) and likely activated its transcription (Fig. 3A). Similarly, the expression of SACE_3900 was upregulated in O*glnR* at 36 h and 72 h by 8- and 5.3-fold respectively, when compared to WT. Conversely, Δ*glnR* showed reduced SACE_3900 expression compared with the WT strain (Fig. 3B). Moreover, the Δ*glnR*::*glnR* strain restored the phenotype to WT levels. These results indicated that PhoP and GlnR positively induced SACE_3900 expression under phosphate and nitrogen limited conditions, respectively.

### PhoP and GlnR increase intracellular SAM content

**Fig. 4:**
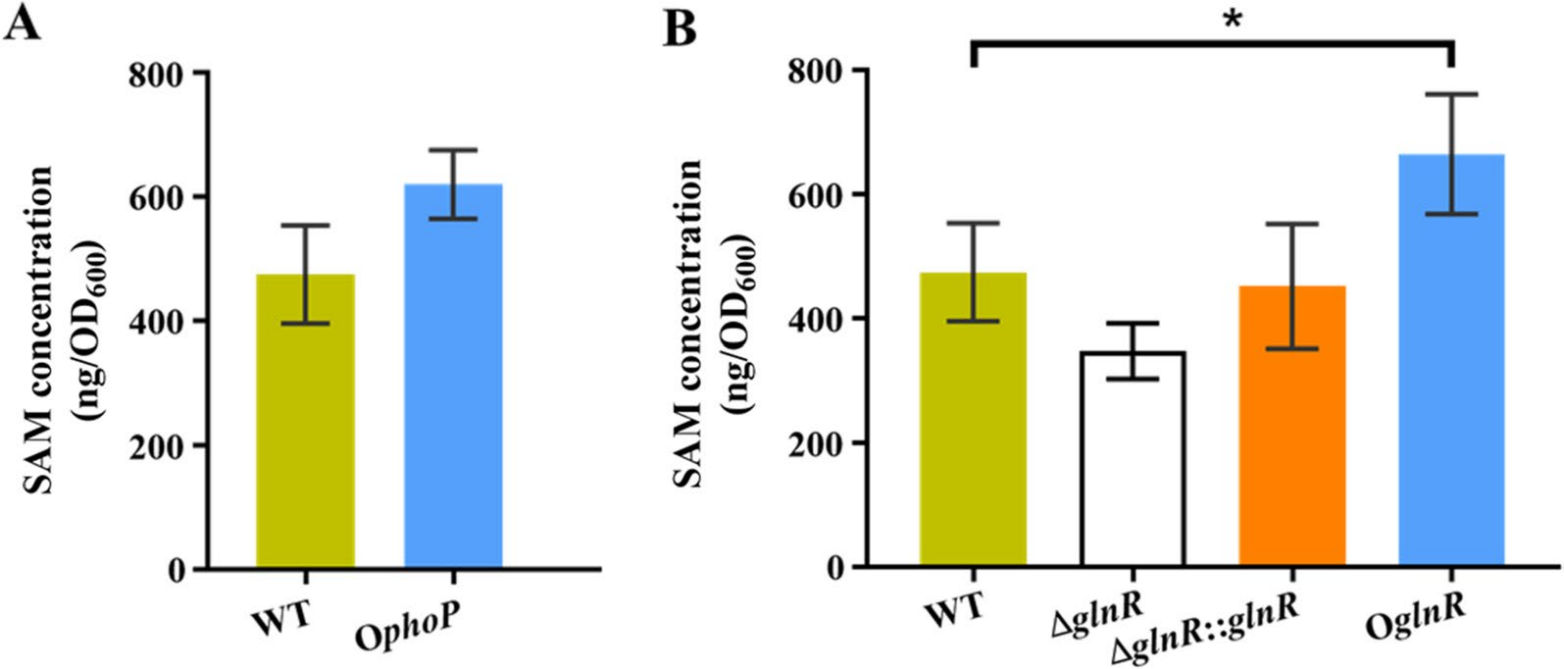
PhoP and GlnR promote SAM production in *S. erythraea*. **A** Content of SAM in WT and O*phoP* strains. **B** Content of SAM in WT, Δ*glnR*, Δ*glnR*::*glnR* and O*glnR* strains. The WT and O*phoP* were grown in phosphate-limiting Evans, while WT, Δ*glnR*, Δ*glnR*::*glnR* and O*glnR* were grown in nitrogen-limiting Evans. Error bars indicate standard deviations from three independent biological replicates. *P < 0.05 by One-way ANOVA and Tukey’s multiple comparisons test using GraphPad Prism 8

Overexpression of *phoP* and *glnR* induced the expression of SACE_3900 in liquid culture (Fig. 3). Next, we determined the intracellular SAM levels of the strains using HPLC. As shown in Fig. 4, the production of SAM was increased in the O*phoP* and O*glnR* strains. The SAM levels in O*phoP* and O*glnR* strains were 1.4- and 1.5-fold (P < 0.05) higher than those in the WT strains, respectively (Fig. 4A, B). In contrast, the Δ*glnR* strain resulted in the downregulation of the SAM concentration compared with the WT strain (Fig. 4B), while the Δ*glnR*::*glnR* strain restored the WT level. The results suggested that the global regulators PhoP and GlnR influenced the SAM content, which is consistent with the influence of each protein upon SACE_3900 transcription levels.

### Effect of GlnR and PhoP on the transformation of Er C to Er A

**Fig. 5:**
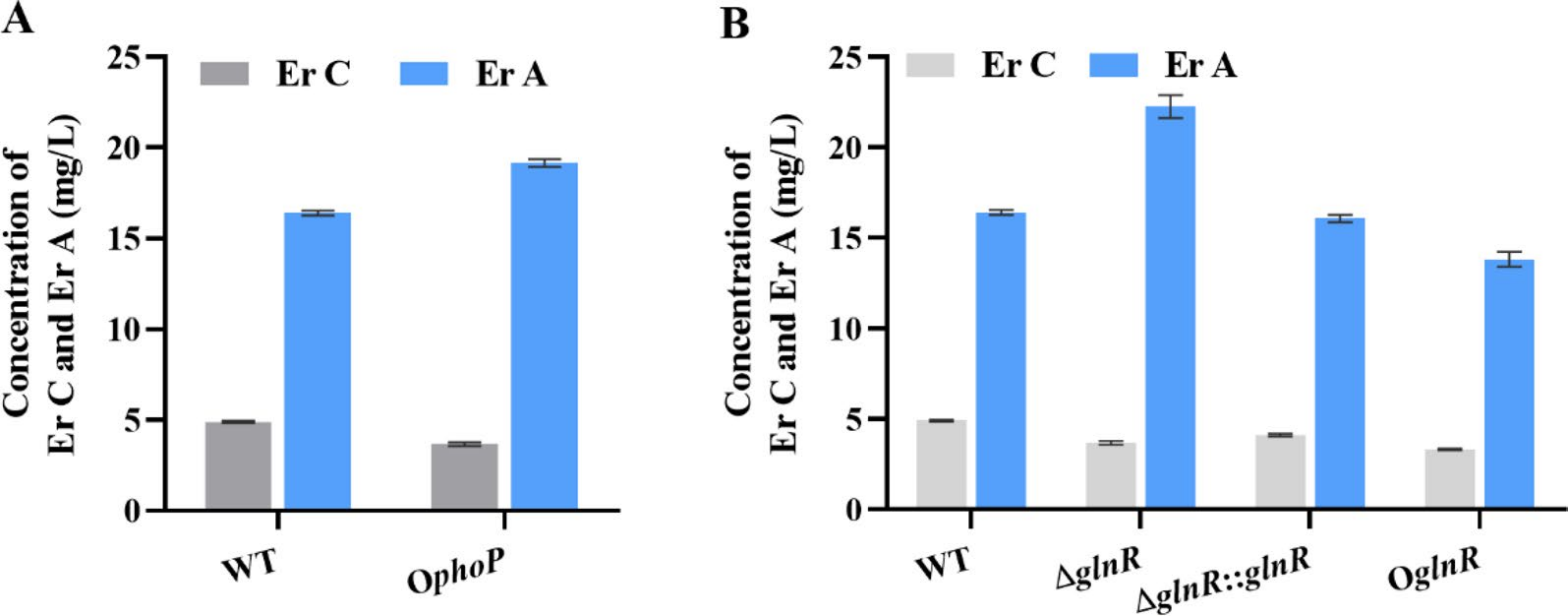
Quantification of Er C and Er A in different *S. erythraea* strains. **A** The concentration of Er C and Er A produced by the WT strain and O*phoP* was measured using HPLC. **B** Er C and Er A concentration of WT, Δ*glnR*, Δ*glnR*::*glnR*, and O*glnR* strains. Strains **A** and **B** grown in phosphate or nitrogen limited medium. Supernatants were harvested after being grown for 6 days. Three independent replicates were operated to calculate mean and the standard deviations

Previous studies have demonstrated that the PhoP response to phosphate starvation induces the expression of the *ery* cluster and further promotes Er A production [19]. Moreover, overexpression of *metK*, *eryK*, and *eryG* significantly increased Er A content and decreased the Er B component in *S. erythraea* [3]. Since it has been shown that GlnR and PhoP could regulate and promote the expression of the putative *metK* gene SACE_3900 (Figs. 1, 2 and 3), we further investigated the relative concentrations of Er C and Er A, and examined the regulation of methylation by GlnR and PhoP from the perspective of the transformation of Er C to Er A, which depends on SAM (coded for by *metK*). The Er A produced by O*phoP* strains increased by 17% compared with that of the WT strain (Fig. 5A). Moreover, the conversion ratio of Er C to Er A increased by about 10%, which further indicated that PhoP promoted Er A production via the methylation of Er C, and this was possibly due to upregulation of *metK*, which is consistent with previously reported studies [19]. However, no significant improvement in Er A production and Er C conversion was observed in O*glnR* strains (Fig. 5B), and surprisingly Er A production in the Δ*glnR* strain was significantly improved by 31% (P < 0.05), and the conversion from Er C to Er A increased by 13% compared with the WT strain. Although the intracellular SAM level was increased in O*glnR*, Er A production and biotransformation from Er C to Er A did not increase. The influence of GlnR on erythromycin biosynthesis promotes the expression of *metK* (Figs. 1, 2 and 3); however, it may also inhibit the erythromycin biosynthesis [19]. Many studies have reported that erythromycin production and biosynthetic gene expression are strongly inhibited by ammonium in over-producing and low-producing *S. erythraea* strains [29]. Since GlnR is a global factor related to nitrogen induction, its detailed mechanism for SAM and erythromycin biosynthesis is worthy of further investigation in subsequent studies. In summary, PhoP and GlnR activate the expression of SACE_3900 and promote SAM production likely via sensing phosphorus and nitrogen signal changes. Only O*phoP* strains activated the production of Er A and conversion of Er C to Er A.

## Discussion

**Fig. 6:**
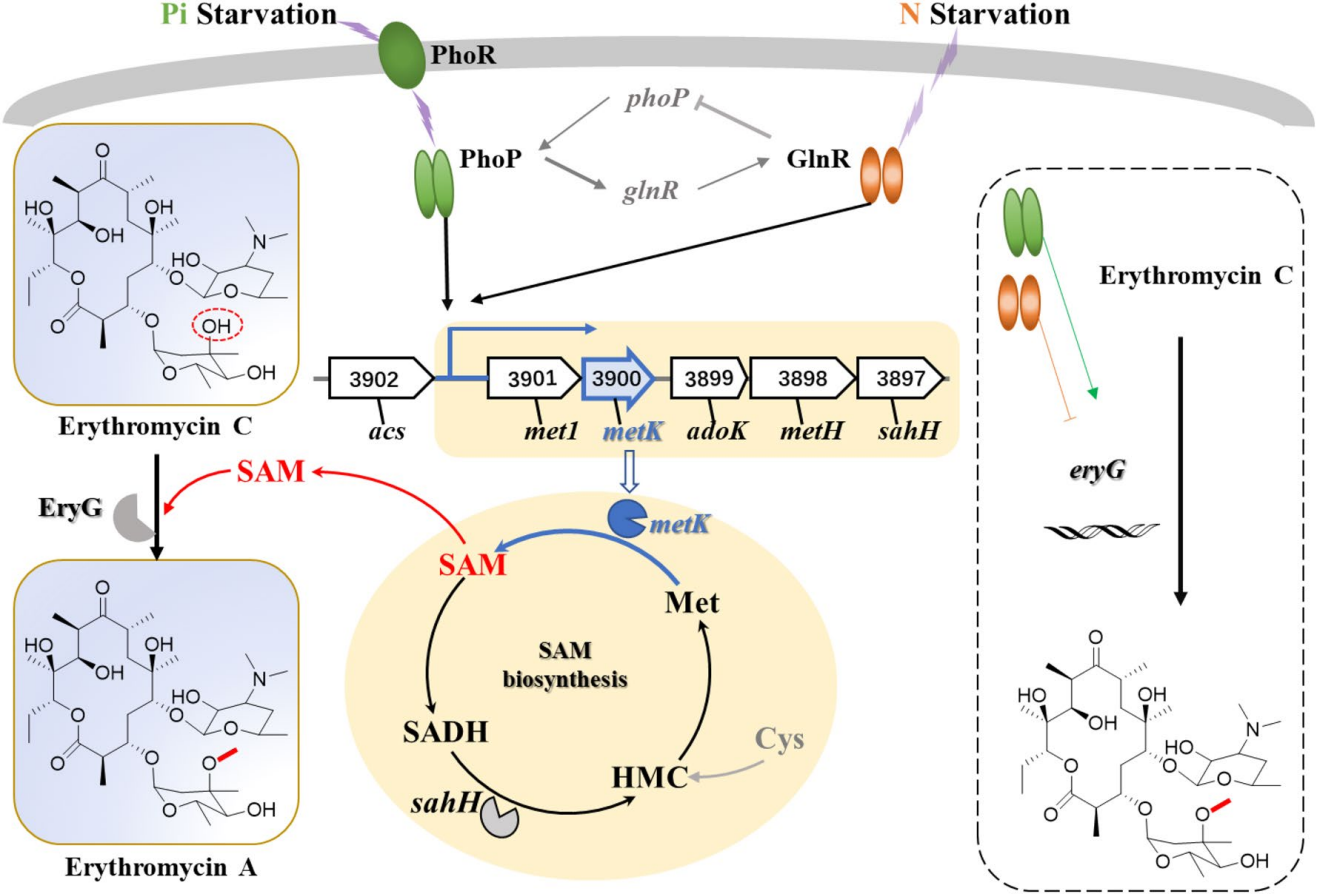
Regulatory model of *metK* mediated by PhoP and GlnR in *S. erythraea*. Solid lines indicate transcriptional regulation. Arrows represent positive influence. Lines represent negative control. S-adenosyl-L-homocysteine, SADH;  homocysteine, HMC; methionine, Met; S-adenosyl-methionine, SAM; cysteine, Cys.

It has been previously reported that the phosphate regulator PhoP plays a vital role in primary and secondary metabolite biosynthesis [2]. The PhoP sensing signal changes through its pairing with the response regulator PhoR, and it regulates gene expression, often via direct binding to the promoter region of target genes [19]. For example, PhoP-*ery* and PhoP-BldD-*ery* nutrient-sensing signal transduction routes have been reported for the regulation of erythromycin biosynthesis [19]. Moreover, the methylation of Er C is the key final step in Er A biosynthesis. Importantly, the methylation process is SAM dose-dependent. Therefore, exogenous SAM or overexpression of *metK* genes should increase Er A production. We speculated that manipulating PhoP to overexpress *metK* might be a new strategy to promote the synthesis of SAM. Studies have also shown a pleiotropic regulatory relationship between PhoP and GlnR (Fig. 6) [19]. The crosstalk between the systems makes it possible for GlnR to regulate the synthesis of erythromycin and SAM. Interestingly, GlnR is a nitrogen regulator has also been shown to have a significant impact on the *ery* gene cluster [29]. Research has shown that erythromycin high-producing strains display lower expression levels of the *glnR* gene than the WT strain [19, 29, 30]. Moreover, erythromycin production was shown to be repressed by ammonium concentration in growth medium [20, 31]. Our study confirmed that the nitrogen regulator GlnR positively regulates *metK* expression (Fig. 6), however it did not ultimately increase the yield of Er A.

The methyl donor SAM participates in the methylation modification of the side chain of antibiotics under the action of methyltransferases, such as EryG [19, 29, 30]. It is known that the methyltransferase-encoding gene *eryG* is directly or indirectly regulated by PhoP and GlnR [19], yet the regulatory mechanism of PhoP and GlnR in SAM synthesis is unknown. In addition to *metK*, shown to be involved in this study, the SAM synthesis cycle also requires the supply of important amino acids, such as cysteine and methionine, as precursors (Fig. 6). Cells can acquire such amino acids by absorption or de novo synthesis and it is well known that GlnR and PhoP in Streptomyces [32–35] regulate the absorption and synthesis of amino acids. Therefore, for follow-up research, the effect of GlnR and PhoP on the transport and synthesis of cysteine and methionine should be investigated. GlnR and PhoP-mediated nitrogen and phosphorus metabolism processes respond to changes in the nitrogen source [20, 36] and phosphorus [37, 38] in the culture medium; conversely, in the anti-biosynthesis approach, the concentration of nitrogen and phosphorus sources can be adjusted to intervene with the functions of GlnR [29, 39] and PhoP [19, 23]. As a result, GlnR and PhoP-mediated regulation for chemical synthesis can be achieved, especially in the context of antibiotic methylation and the regulation of precursor SAM supply. From the perspective of metabolic engineering, through overexpression or deletion of global regulatory factors PhoP and GlnR, the conversion of antibiotic precursors to final products is achieved. The above research will promote the development of SAM-dependent antibiotic synthesis, and the discovery of efficient strategies for the synthesis and transformation of methylated antibiotics.

## Materials and methods

### Strains, plasmids, and culture conditions

Strains and plasmids mentioned in this experiment are shown in Table 1. *S. erythraea* strains were grown in TSB or Evans media. Evans media were used as described previously [19, 30], including phosphate-limiting Evans (40 μM NaH_2_PO_4_ and basic C and N), nitrogen-limiting Evans [1 mM (NH_4_)_2_SO_4_ and basic C and P) and basic Evans [7.5 mM (NH_4_)_2_SO_4_, 8 mM NaH_2_PO_4_] medium with 1% glucose. *Escherichia coli* was grown in Luria–Bertani (LB) liquid media or on LB plates at 37 °C. All media were autoclaved at 121 °C for 20 min. Glucose was autoclaved at 115 °C for 30 min and then added to Evans medium.

**Table 1:** Strains and plasmids used in this study

Strain or plasmid	Relevant characteristic (s)	Source or references
Strains		
*S. erythraea* NRRL2338	Wild-type strain	DSM
Δ*glnR*	*glnR* knockout strain	[20]
O*glnR*	*glnR* overexpression strain	[30]
O*phoP*	*phoP* overexpression strain	[2]
Δ*glnR::glnR*	*glnR* complementary strain	[30]
*E. coli*		
DH5α	F¯Ф80*dlacZ*ΔM(*lacZYA-argF*)U169 *deoR*	GIBCO-BRL
BL21(DE3)	F′*ompT hsdS gal dcm* (DE3)	Novagen
Plasmids		
pET-28a	Expression vector with T7 promoter	Novagen
pET-*glnR*	pET28a with *glnR*	[20]
pET-*phoP*	pET28a with *phop*	[2]
pIB139	*E. coli*-*S. erythraea* integrative shuttle vector containing *ermE* promoter	Lab stock
pIB*-glnR*	pIB139 with *glnR* for gene overexpression	Lab stock
pIB*-phoP*	pIB139 with *phoP* for gene overexpression	Lab stock

### Computational analysis

The predicted PhoP and GlnR binding box in the upstream promoter regions of *metK *genes were identified using the MEME/MAST tool and PREDetector software program. The details of the prediction methods have been described previously [40].

### Electrophoretic mobility shift assay (EMSAs)

Predicted promoters of putative *metK* genes (SACE_2103 and SACE_3900) were amplified by PCR using gene-specific and 5′-biotin-labeled universal primers (Table 2). PCR probes were verified using agarose gel electrophoresis and purified with a PCR purification kit (Beijing TIANGEN Biotech Co., Ltd., China). The probe concentration was measured using a microplate reader (Biotek, USA). EMSAs were performed as described by Xu et al. [19]. The samples were separated on a 6% non-denaturing PAGE gel in an ice-bath of 0.5 × Tris–borate-EDTA at 120 V, and band shifts were tested by BeyoECL P.

**Table 2:** Oligonucleotides used in this study

Oligonucleotide	Sequence (5′–3′)^*a*^
Primers for construction Δ*glnR* strain	
F-*glnR*-up	CCC*AAGCTT*CACCGGCGATGTTGACCGACCCGTC
R-*glnR*-up	GC*TCTAGA*GAGGAGGGCCTCCATCCCAGGGCGG
F-*glnR*-dw	CGG*GGTACC*GTTCGAAGCGTGCAGCTCACCTGG
R-*glnR*-dw	CCG*GAATTC*GCGAGTCCGAGCCGCCGAAGTCGAT
Primers for construction O*glnR* strain	
F-O*glnR*	GGAATTC*CATATG*ATGAGCTCTGAGCTTCTCCTGCTC
R-O*glnR*	TGCAG*GATATC*TCAGCGGACGACCGCGG
Primers for construction O*phoP* strain	
F-O*phoP*	GGAATTC*CATATG*GTGACCAGGGTGCTGATCGTG
R-O*phoP*	GAC*GATATC*CTACACCTCGAACTTGTAGCCGA
Primers for biotin labeling EMSAs probe	
Bio-primer	Biotin-AGCCAGTGGCGATAAG
Primers for EMSAs	
SACE_2103F	AGCCAGTGGCGATAAGCCGCAAGGCCTGCGAC
SACE_2103R	AGCCAGTGGCGATAAGGCTGATCGCGTCGCAGATC
SACE_3900F	AGCCAGTGGCGATAAGAGACCGTGGACTTCGAGGATCTCAG
SACE_3900R	AGCCAGTGGCGATAAGATGGAGTCGCTGATCGCGTCG
SACE_3900-1F	AGCCAGTGGCGATAAGAGACCGTGGACTTCGAGGATCTCAGCG
SACE_3900-1R	AGCCAGTGGCGATAAGATGCGGTCGGAGAGCTCCTGTTCG
SACE_3900-2F	AGCCAGTGGCGATAAGCGGCGAGGATTACCGGAACCGG
SACE_3900-2R	AGCCAGTGGCGATAAGAGACTGCGCATGGAAGATCCCCCAC
SACE_3900-3F	AGCCAGTGGCGATAAGTGACAGTGACCGTCCGTACGCAAGGATCG
SACE_3900-3R	AGCCAGTGGCGATAAGCGACTCACTCGTGAACAACCTGCGGTTGATC
Primers for PCR	
RT6965-F	AGCGGGACCGACGTCTGC
RT6965-R	TGACCTCCTCGCCGGAGAC
RT7101-F	CGCCGAGTGGGGTGTGG
RT7101-R	CCGAAGAAGTCGTAGCCCCAG
RT3900-F	GTCATCGACGAGATCGCCAAGCAG
RT3900-R	GTGCTGGGTGGACAGCACCACC

### RNA preparation and quantitative reverse transcription polymerase chain reaction (RT-qPCR)

WT and mutant strains were all activated in TSB culture for 48 h at 30 °C and then transferred into phosphate- or nitrogen-limiting Evans media. Cells were collected by centrifugation (8000 rpm for 10 min at 4 °C) at 36 and 72 h and were washed with saline thrice. RNA preparation methods and integrity analysis were performed as described by Xu et al. [2]. RNA concentration was measured using a microplate reader (BioTek). cDNA was synthesized using a PrimeScript reverse transcription (RT) reagent kit with gDNA Eraser (TIANGEN, Beijing). The primers used for qRT-PCR are shown in Table 2. The qRT-PCR experiments were executed using a StepOne plus real-time system (ABI, CA), and the reaction conditions were described by Xu et al. [19]. The 16S rRNA gene was used for reference, and variations in transcription were calculated using the reported method [19].

### SAM (S-adenosyl-methionine) determination

WT and O*phop* were grown in phosphate-limited Evans medium, whereas WT, Δ*glnR*, Δ*glnR*::*glnR*, and O*glnR* were grown in nitrogen-limited Evans medium. Cells were harvested by centrifugation (8000 rpm, 10 min, 4 °C), followed by incubation with 20 mL 10% trichloroacetic acid for 2 h at 25 °C with continuous stirring. The supernatant was collected (8000 rpm, 4 °C) and freeze-dried. The dried samples were then dissolved in 1 mL of sterile water. Intracellular SAM content was determined by high-performance liquid chromatography (HPLC) as described by Payne [41]. Dissolved samples were first obtained through a 0.22-μm pore-size filter and tested with a Hypersil BDS-C18, 5-μm, 4.6 × 250 mm column (Agilent HP1260). The testing conditions were shown below: mobile phase [(10 mM HCOONH_4_): C_2_H_3_N 1: 9]  and detection wavelength of 254 nm (UV–VIS).

### Fermentation and erythromycin determination

Fermentation (50 mL) was harvested by centrifugation (8000 rpm*,* 10 min, 4 °C), and the supernatant was freeze-dried. Dried samples were dissolved in 1.5 mL acetonitrile through a 0.22-μm pore-size filter and tested with HPLC. The testing conditions were as follows: mobile phase [(50 mM K_2_HPO_4_; pH 6.8): C_2_H_3_N 60:40], detection wavelength (215 nm), chromatographic column (5 μm, 4.6 × 250 mm), and rate (1 mL min^−1^) [19].

## Supplementary Information

**Additional file 1: Figure S1.** The sequence alignment of two *metK* in *S. erythraea*. **Figure S2.** The sequence of upstream promoter region and putative PhoP and GlnR binding sites of SACE_3900. **Figure S3.** **A** Growth curve of *S. erythraea* WT, O*phoP* strains grown in phosphate-limiting medium and **B** WT, Δ*glnR*, Δ*glnR*:: *glnR*, O*glnR* grown in nitrogen-limiting medium.

## Data Availability

All data generated or analysed during this study are included in this published article.
