# Supplementary material for: PhoP- and GlnR-mediated regulation of metK transcription and its impact upon S-adenosyl-methionine biosynthesis in Saccharopolyspora erythraea
Source: Microb Cell Fact. 2022 Jun 18;21:120. doi: 10.1186/s12934-022-01846-w (PMC9206729; doi:10.1186/s12934-022-01846-w)
Supplement: Supplementary file 1 — Additional file 1: Figure S1. The sequence alignment of two metK in S. erythraea. Figure S2. The sequence of upstream promoter region and putative PhoP and GlnR binding sites of SACE_3900. Figure S3. A Growth curve of S. erythraea WT, OphoP strains grown in phosphate-limiting medium and B WT, ΔglnR, ΔglnR:: glnR, OglnR grown in nitrogen-limiting medium. [file 12934_2022_1846_MOESM1_ESM.docx]

**Supporting Information**

PhoP- and GlnR-mediated regulation of *metK* transcription and its impact upon S-adenosyl-methionine biosynthesis in *Saccharopolyspora erythraea*


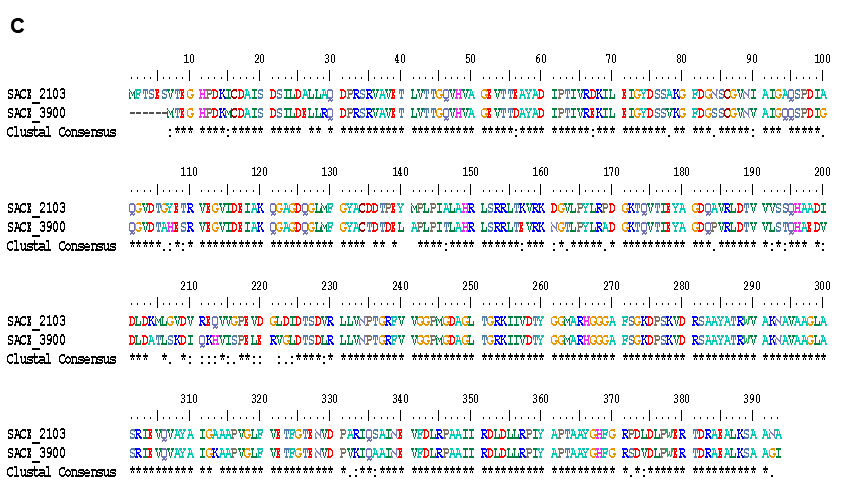


**Figure S1**. The sequence alignment of two *metK* in *S. erythraea*


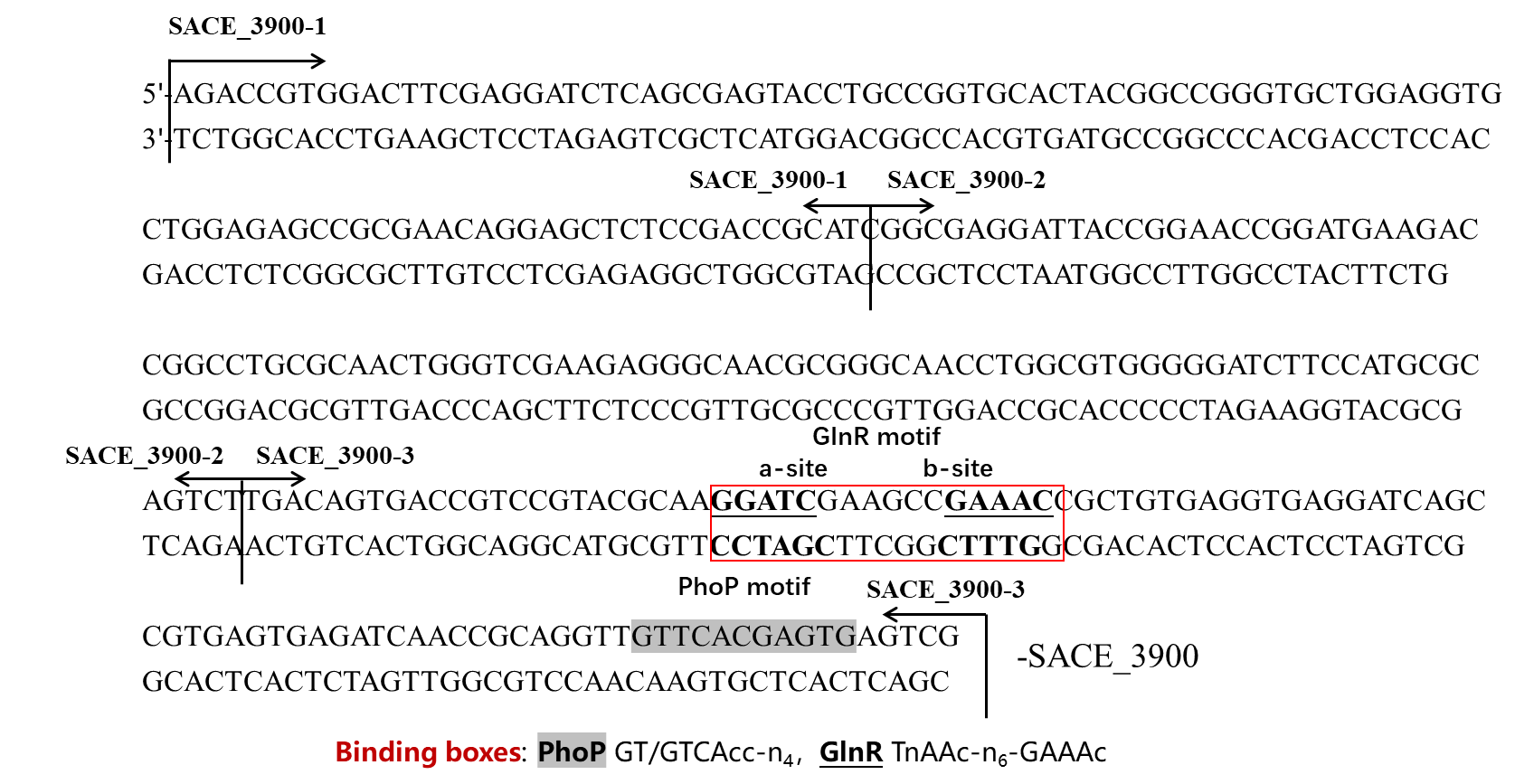


**Figure S2**. The sequence of upstream promoter region and putative PhoP and GlnR binding sites of SACE_3900.


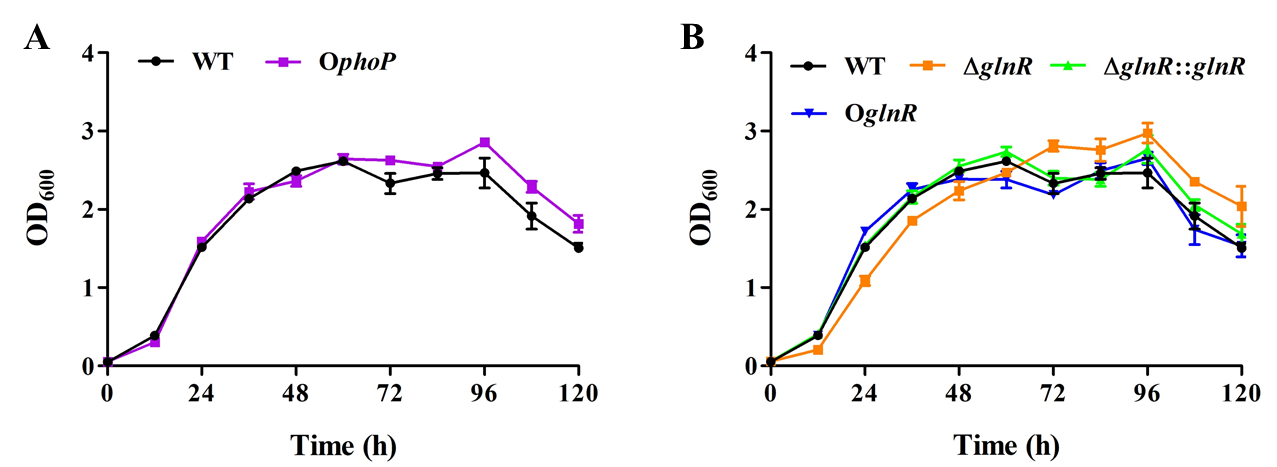


**Figure S3**. (A) Growth curve of *S. erythraea* WT, O*phoP* strains grown in phosphate-limiting medium and (B) WT, Δ*glnR*, Δ*glnR*:: *glnR*, O*glnR* grown in nitrogen-limiting medium.
